# Association of Genetic Variants with Self-Assessed Color Categories in Brazilians

**DOI:** 10.1371/journal.pone.0083926

**Published:** 2014-01-08

**Authors:** Danielle Fernandes Durso, Sergio Paulo Bydlowski, Mara Helena Hutz, Guilherme Suarez-Kurtz, Tiago R. Magalhães, Sérgio Danilo Junho Pena

**Affiliations:** 1 Departamento de Bioquímica e Imunologia, Universidade Federal de Minas Gerais, Belo Horizonte, MG, Brazil; 2 Laboratório de Genética e Hematologia Molecular, Faculdade de Medicina, Universidade de São Paulo, São Paulo, SP, Brazil; 3 Departamento de Genética, Universidade Federal do Rio Grande do Sul, Porto Alegre, RS, Brazil; 4 Coordenação de Pesquisa/Divisão de Farmacologia, Instituto Nacional do Câncer (INCA), Rio de Janeiro, RJ, Brazil; 5 Laboratório de Genômica Clínica, Faculdade de Medicina, Universidade Federal de Minas Gerais, Belo Horizonte, MG, Brazil; University of Florence, Italy

## Abstract

The Brazilian population was formed by extensive admixture of three different ancestral roots: Amerindians, Europeans and Africans. Our previous work has shown that at an individual level, ancestry, as estimated using molecular markers, was a poor predictor of color in Brazilians. We now investigate if SNPs known to be associated with human skin pigmentation can be used to predict color in Brazilians. For that, we studied the association of fifteen SNPs, previously known to be linked with skin color, in 243 unrelated Brazilian individuals self-identified as White, Browns or Blacks from Rio de Janeiro and 212 unrelated Brazilian individuals self-identified as White or Blacks from São Paulo. The significance of association of SNP genotypes with self-assessed color was evaluated using partial regression analysis. After controlling for ancestry estimates as covariates, only four SNPs remained significantly associated with skin pigmentation: rs1426654 and rs2555364 within *SLC24A5*, rs16891982 at *SLC45A2* and rs1042602 at *TYR*. These loci are known to be involved in melanin synthesis or transport of melanosomes. We found that neither genotypes of these SNPs, nor their combination with biogeographical ancestry in principal component analysis, could predict self-assessed color in Brazilians at an individual level. However, significant correlations did emerge at group level, demonstrating that even though elements other than skin, eye and hair pigmentation do influence self-assessed color in Brazilians, the sociological act of self-classification is still substantially dependent of genotype at these four SNPs.

## Introduction

Brazilians form one of the most heterogeneous populations in the world, the result of five centuries of interethnic crosses of peoples from three continents: the European colonizers, the African slaves, and the autochthonous Amerindians. The relative proportion of these three ancestral roots in the makeup of the Brazilian population has changed considerably along time. After more than 100 years of heavy European immigration beginning in the second half of the 19^th^ Century, all regions of Brazil now show a preponderance of European ancestry, with proportions ranging from 60.6% in the Northeast to 77.7% in the South [1].

In Brazil, color (in Portuguese, *cor*) is based on a complex phenotypic evaluation that takes into account not only skin pigmentation, but also hair pigmentation and type, eye melanization and facial features such as nose and lip shape [2], [3]. Since 1991, the Instituto Brasileiro de Geografia e Estatística (IBGE), responsible for the official census of Brazil, has employed only five pre-established discontinuous color categories, exclusively based on self-assessment: White, Browns, Blacks, Yellows, and Indigenous. In 2010, the IBGE census computed a population of 191 million Brazilians, into the following color percentages: 47.6% White, 43.0% Brown, 7.6% Black, 0.6% Yellow, 1.0% Indigenous, and 0.1% with no declaration (http://www.sidra.ibge.gov.br/bda/tabela/listabl.asp?z=cd&o=4&i=P&c=3145). Justifying this strategy, it has been shown that even when there is total liberty for the declaration of “color or race” without a priori definition of categories, most Brazilians identify themselves spontaneously according to this relatively restricted group of color representations [4], [5].

Our previous studies [1], [6]–[8] have shown that biogeographical ancestry is a poor predictor of color in Brazil. Thus, we decided to ascertain whether SNPs that have been shown to be associated with skin, eye and hair pigmentation in other populations could perform better in the prediction of self-declared color in Brazilians. For this purpose, we analyzed 15 such SNPs in 455 unrelated Brazilian individuals self-declared as Whites, Browns or Blacks, from two large cities in the Southeast of Brazil. We here report our results in this study.

## Results

### Association of self-classification of color with genotypes at SNPs known to influence pigmentation in Rio de Janeiro

Our main target population was composed of 243 unrelated Brazilian individuals from the city of Rio de Janeiro, self-evaluated as Whites (n = 82), Browns (n = 80) or Blacks (n = 81), according to the census criteria of IBGE. As mentioned in the Introduction, our previous studies [1], [6]–[8], have shown that geographical ancestry is not a good predictor of color in Brazil. Thus, we initially tested if the same observation was valid for this specific group.

**Figure 1:**
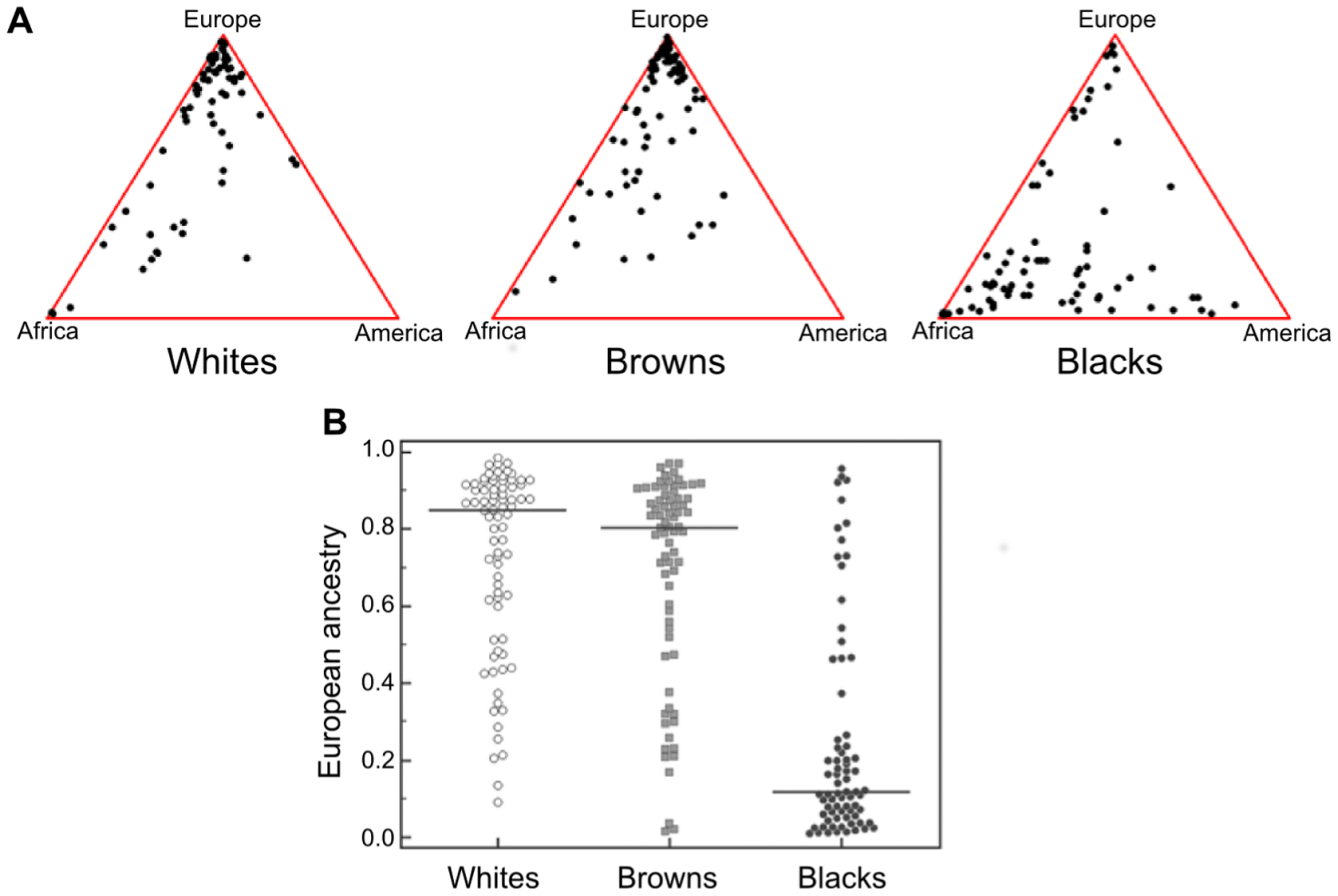
(A) Triangular plots of the genomic proportions of African, European and Amerindian ancestry in three self-reported color groups of 243 Brazilian individuals from Rio de Janeiro samples, self-categorized as White, Brown and Black individuals. Each point represents a separate individual and the ancestral proportions can be determined by dropping lines parallel to each of the three axes. The graphs were drawn using the *Tri-Plot* software. (B) Dot plot of the European ancestry proportion for each separate self-assessed color group.

To achieve that, as previously done [1], [8], [9], we genotyped all individuals at 40 autosomal short insertion-deletion polymorphisms (indels) dispersed in the human genome. We then used the genotypes and the *Structure* program [10] to estimate, at an individual level, the European, African and Amerindian components of ancestry. All the individual estimates are shown in triangular plots for each separate self-assessed color group in Figure 1A.

To provide a clearer perspective, we also display in Figure 1B the dot plot of the European ancestry proportion for each separate self-assessed color group. Whites had a median proportion of European ancestry of 0.849 and an Inter-Quantile Range (IQR = 75^th^ percentile – 25^th^ percentile) of 0.336; Browns had a median of 0.803 and IQR of 0.353; Blacks had a median of 0.119 and IQR of 0.210. From inspection of these images it is evident that the color groups had very wide variance and that there was very significant overlap between them, making it impossible to confidently predict color from ancestry at an individual level, the distinction between Whites and Browns being especially difficult. On the other hand, if we assign to the White, Brown and Black categories numerical values 0.0, 0.5 and 1.0, respectively, we obtain a Spearman's rank correlation rho value of −0.557, which is significant (P<0.0001).

### Association of self-classification of color with genotypes at SNPs known to influence pigmentation in Rio de Janeiro

We next genotyped all 243 unrelated individuals from the city of Rio de Janeiro, for the 15 SNPs known to be associated with pigmentation of skin, eyes and/or hair (Table 1) using the *Taqman* SNP genotyping assays (Table S1 in File S1). Allele frequencies for all loci are shown in Table S2 in File S1.

**Table 1:** Numeric regression analysis between self-assessed color categories and SNP genotypes in population samples from Rio de Janeiro and São Paulo.

	Rio de Janeiro		São Paulo	
RefSNP	NR	CV	NR	CV
rs26722	1.000E+00	1.000E+00	-	-
rs642742	1.610E-01	1.000E+00	7.851E-19^*^	1.562E-04^*^
rs1015362	8.446E-03^*^	1.000E+00	4.773E-08^*^	1.657E-02^*^
rs1042602	3.268E-06^*^	3.606E-02^*^	4.389E-12^*^	5.950E-04^*^
rs1126809	1.000E+00	1.000E+00	-	-
rs1408799	1.943E-01	1.000E+00	-	-
rs1426654	4.032E-20^*^	2.080E-09^*^	2.350E-46^*^	9.399E-20^*^
rs1800401	1.000E+00	1.000E+00	-	-
rs1800407	1.000E+00	1.000E+00	-	-
rs2555234	6.124E-13^*^	6.660E-07^*^	3.024E-22^*^	8.123E-08^*^
rs2733832	3.187E-02^*^	8.883E-01	1.081E-08^*^	1.042E-03^*^
rs6058017	2.874E-05^*^	5.349E-02	1.365E-10^*^	4.543E-02^*^
rs12896399	5.239E-02	1.000E+00	4.712E-02^*^	1.000E+00
rs12913832	1.038E-01	1.000E+00	1.269E-07^*^	4.879E-03^*^
rs16891982	4.511E-17^*^	3.460E-09^*^	3.550E-37^*^	6.787E-17^*^
Amerindian ancestry	4.612E-04^*^	-	1.223E-02^*^	-
African ancestry	2.588E-13^*^	-	1.761E-37^*^	-
European ancestry	8.234E-17^*^	-	1.757E-41^*^	-

NR - Numeric full model regression.

CV - restricted model (partial correlation with ancestry as a covariate). Significant values (<0.05) after Bonferroni correction are shown with an asterisk.

To perform a statistical association analysis using numeric regression, we then converted, as above, the self-classified color (White, Brown, and Black) into the following numeric values: 0.0, 0.5 and 1.0 respectively. In this fashion, we could use the Golden Helix SVS7 software to perform a numeric regression of phenotypes on genotypes, which had also been converted to numeric values under three models: co-dominant (additive), dominant and recessive models. The additive model presented the lowest P values (Table S3 in File S1) and was adopted for all further analyses. After application of the Bonferroni correction for multiple comparisons, we observed that, as shown in Table 1, seven loci showed P values under the 5% significance level (rs1426654, rs16891982, rs2555364, rs1042602, rs6058017, rs1015362 and rs2733832, in decreasing order of significance). As shown previously, self-assessed color showed significant associations with the estimated proportions of European, African and Amerindian ancestries at group level.

To eliminate ancestry confounding we used the Golden Helix SVS7 software to perform a partial regression analysis using European, African and Amerindian ancestry estimates as covariates. After applying that ancestry control, only four SNPs remained with significant association: rs1426654 and rs2555364 within locus *SLC24A5*, rs16891982 at locus *SLC45A2*, and rs1042602 at locus *TYR*.

### Association of self-classification of color with genotypes at SNPs known to influence pigmentation in São Paulo

To ascertain whether these four loci could be confirmed as the most significant in a different Brazilian population, we evaluated 212 unrelated individuals from the city São Paulo, which, as Rio de Janeiro, is also located in Southeastern Brazil. However, this sample differed from the one from Rio de Janeiro in that it was made up only of individuals self-evaluated as Whites (n = 106) or Blacks (n = 106), thus missing individuals self-classified as Browns. Moreover, individuals from São Paulo were only tested for ten SNPs from the 15 SNPs originally tested in Rio de Janeiro, but included, of course, all seven found to be significantly associated with self-classified color on our full-model numeric regression.

To be able to perform numerical analysis we then converted the self-classified color (White and Black) into the numeric values 0.0 and 1.0 respectively, and used the Golden Helix SVS7 software to regress color phenotypes on genotypes, also converted to numeric values, under the co-dominant (additive) model. After application of the Bonferroni correction for multiple comparisons, we observed that, as shown in Table 1, all loci showed significance, with P values lower than in the Rio de Janeiro group, presumably because we used only individuals on the polar color groups (Whites and Blacks), without the intermediate Browns.

Since the proportions of European, African and Amerindian ancestries also showed significant associations with color, we again proceeded to control for biogeographical ancestry confounding and obtain an estimate of the importance of self-identified color alone using a partial regression analysis using the ancestry estimates as covariates. After that, nine SNPs remained with significant association at the 0.05 level after the Bonferroni correction (Table 1). The first, second, third and fifth lowest significance (P) values in the in the São Paulo population sample were observed for SNPs rs1426654, rs16891982, rs2555234 and rs1042602, which had all previously been found to be significant in the Rio de Janeiro population (rs2555364-rs1426654 haplotypes on locus *SLC24A5*, rs16891982 on locus *SLC45A2*, and rs1042602 in locus *TYR*). The difference was that SNP rs642742 close to gene *KITLG* now was also significant, while it had not been significant with the Rio de Janeiro sample.

### Linkage disequilibrium of rs2555364 and rs1426654

The SNPs rs2555364 and rs1426654 are on positions 48,419,386 and 48,426,484 on chromosome 15, only 7,098 base pairs apart. Hence, they are expected to be in linkage disequilibrium. Indeed, our analysis of the data using the web tool CUBEX [11] confirmed that, showing D′ values of respectively 1.0, −1.0 and −0.88 for Whites, Browns and Blacks from the Rio de Janeiro sample and D′ values of respectively 1.0 and −0.94 for Whites and Blacks from the São Paulo sample. This meant that for best accurate results, we should use haplotypes at both SNPs, rather than treating their alleles independently. The haplotype frequencies are shown in Table S4 in File S1. We estimated the haplotype phase for each individual we using the *PHASE* program for Windows (http://stephenslab.uchicago.edu/software.html#phase).

### Cluster analysis of the sample groups from Rio de Janeiro and São Paulo using the Structure software

**Figure 2:**
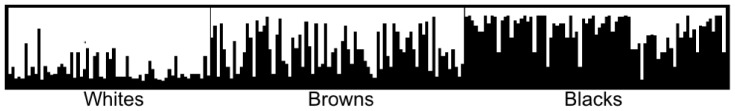
Graph of the individual value of the “Pigmentation Index” (PI) estimated using the Structure software, on the basis of the proportion of belonging to two clusters. Each thin vertical line represents one individual (243 in total). Vertical black lines separate the individuals into three different self-categorized skin color, identified by the labels on the bottom. Ten *Structure* runs were performed with a burn-in of 100,000 iterations and run length of 2,000,000 iterations.

We then used the rs2555364-rs1426654 haplotypes within locus *SLC24A5*, rs16891982 at locus *SLC45A2*, and rs1042602 at locus *TYR* and the Structure software to estimate clusters among the individuals from our target population of Rio de Janeiro (using K = 3). The program assigned to each individual a value, which represented the probability of belonging to each of the three self-classified color clusters. This value increased progressively as we increased in pigmentation, moving from the White group (mean  = 0.22) to the Brown group (mean  = 0.48) to the Black group (mean  = 0.73), as can be seen in Figure 2. Since this probability value assigned by the Structure program was positively correlated with pigmentation, we have called it “Pigmentation Index (PI)”.

**Figure 3:**
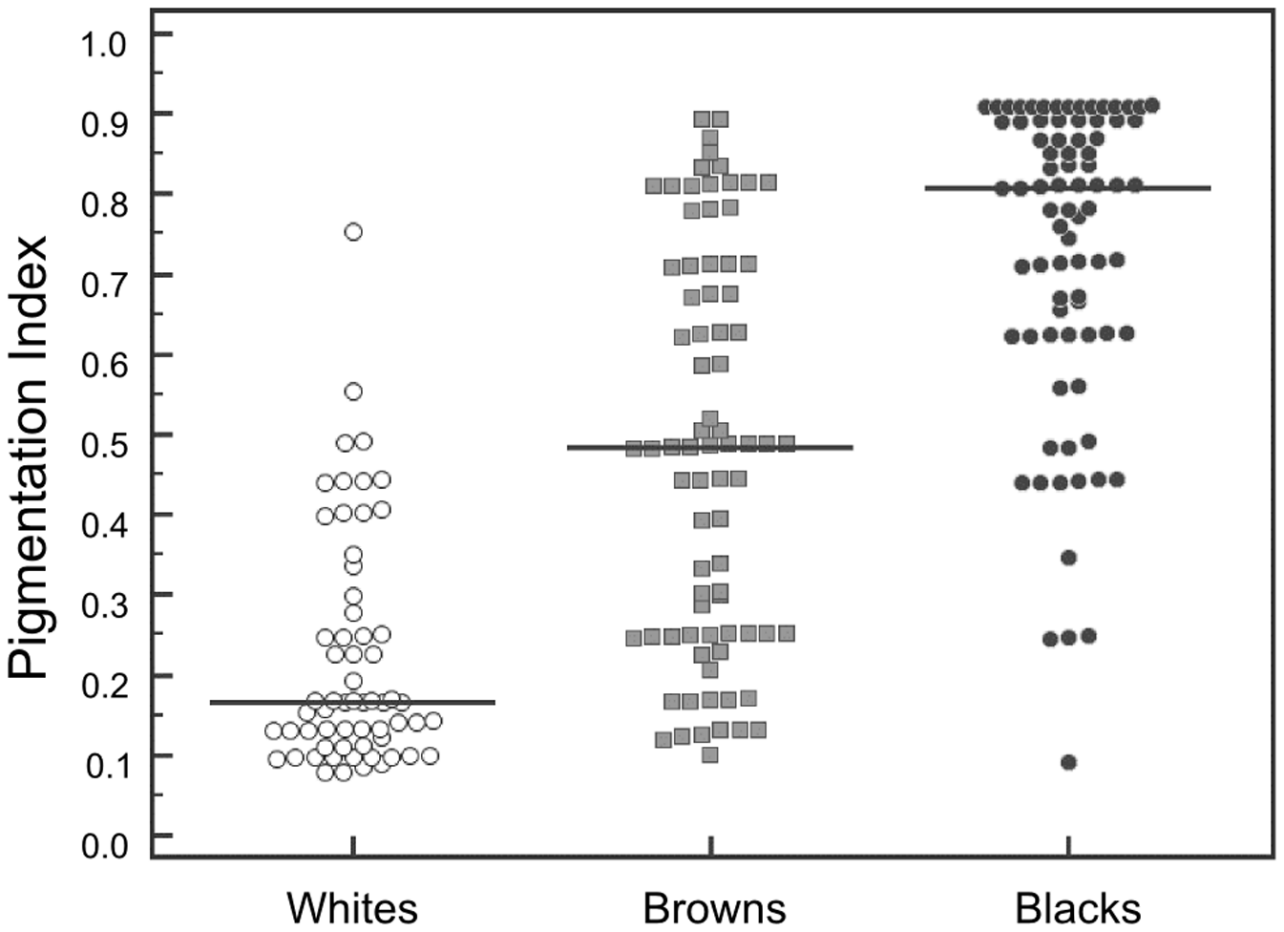
Dot-plot of the “Pigmentation Index” (PI) within the three color categories. The dot-plot was prepared with the MedCalc software [50].

Figure 3 shows the dot plot of PI values for the three color groups of Rio de Janeiro. As observed before for the ancestry estimation, it is evident that that the color groups had wide variance and that there was very significant overlap between them, making it impossible to confidently predict color categories from SNP genotypes at an individual level. Browns showed much more intermediate levels of PI between Whites and Blacks than observed with ancestry.

### Prediction of color from the joint analysis of ancestry and the pigmentation index

From the previous results, emerges a picture in which neither biogeographical ancestry nor the pigmentation index (PI) calculated from the genotypes of rs1426654, rs16891982, rs2555364, rs1042602 were capable of predicting well the self-assessed color group of individuals from Rio de Janeiro, although the second appeared to resolve better the three groups. On the other hand, our results using partial regression demonstrated that the association of rs2555364-rs1426654 haplotypes, rs16891982 and rs1042602 with self-assessed color was, at least in part, independent of ancestry estimates. This statistical independence was made evident by the differences between the distribution of ancestry (Figure 1) and PI (Figure 3) among the different color groups. We then decided to ascertain if the joint consideration of ancestry and PI could provide a better prediction of self-assessed color.

**Figure 4:**
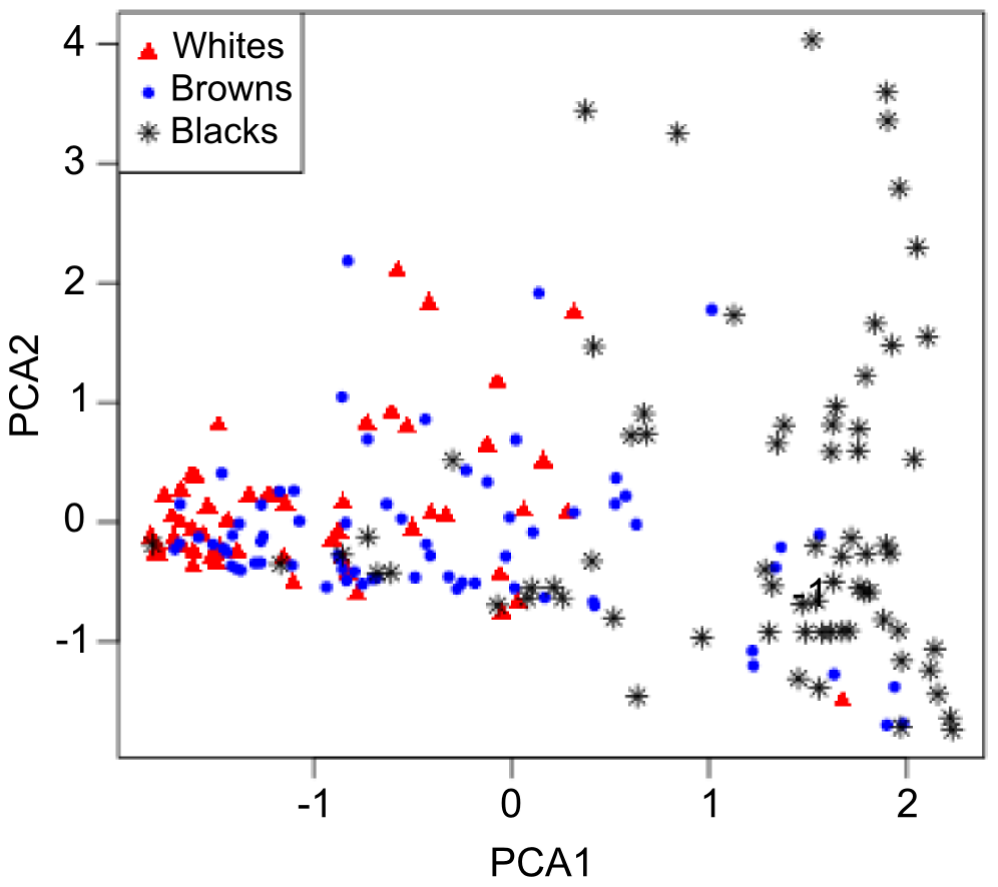
Bi-plot of principal component 1 (PC1) vs. principal component 2 (PC2), with the three color groups shown in different colors and symbols. Each point represents one individual, self-assessed as Whites (red triangles), Browns (blue dots) and Blacks (black asterisks). We ran the Principal Component Analysis (PCA) using three variables: Pigmentation Index (PI), African ancestry and Amerindian ancestry. The PCA and the plot were done using R program, v. 3.0.0 [51].

We tested this hypothesis experimentally using Principal Component Analysis (PCA) with three variables: Pigmentation Index (PI), African ancestry and Amerindian ancestry. The bi-plot of principal component 1 (PC1) vs. principal component 2 (PC2) is shown in Figure 4, with the three color groups shown in different colors and symbols. The first and second components explained 87% (56% and 31%, respectively) of the variance. However, once again, there was no discrete clustering of the three color groups.

## Discussion

### Genetic natures of the associations

On the basis of association studies present in the literature, we chose several genetic variants previously associated with differences in skin, eye and hair pigmentation among individuals from different parts of the world [12]–[17]. In this study, we have demonstrated that after correction for ancestry as a confounding covariate, haplotypes of rs2555364-rs1426654 within *SLC24A5* and the SNPs rs16891982 in *SLC45A2* and rs1042602 in *TYR* are significantly associated with skin color categories in a sample of the population of Rio de Janeiro, with confirmation with a second sample, from São Paulo, another large southeastern city in Brazil.

The regression results were consistent with those of Pneuman et al. (2012) who showed that a set of SNPs containing rs12913832, rs16891982, and rs1426654 was associated with skin color in various populations around the world. Similarly, the SNPs rs1426654 and rs16891982 are known to be related to hair, skin, and eye color of North Americans [18] and individuals of South Asian descent [19]. These findings collectively support the functional role of the loci *SLC24A5* and *SLC45A2* in human pigmentation [20]. The SNP rs1042602 also has been described as associated with pigmentation in general: with eye and hair pigmentation in Europeans [10], [16], [18].

Earlier this year, Beleza et al [21] reported a genome-wide association study of skin color with 879,359 SNPs in an admixed population from Cape Verde. They observed four major loci for skin pigmentation, three of which were exactly the ones that we found significant in our study: *SLC24A5*, *SLC45A2* and *TYR* (the fourth one, *APB2* had not been previously described as associated with pigmentation and thus was not included in the present study). They estimated that these three loci were responsible for 35% of the total variance in color.

*SLC24A5* (solute carrier family 24 member 5) is a well-known human pigmentation locus, which is a human orthologue of the *golden* gene of zebra fish [22], [23]. The cSNP rs1426654 involves an A to G transition on position 48,426.484 of chromosome 15, causing a Thr111Ala mutation, which apparently has an effect in melanosome biogenesis, possibly because of incomplete processing of a protein integral to early melanosome production [24]. The G allele is much abundant in African and Asian populations (Cheng and Canfield, 2006). The non-transcribed SNP rs2555364, involving a C>G transversion, again has much higher frequencies of the G allele in African and Asian populations (http://www.ncbi.nlm.nih.gov/projects/SNP/snp_ref.cgi?rs=2555364). Because of the continental correlation between color and biogeographical ancestry, haplotypes of rs1426654, rs2555364 and a third SNP rs16960620 can be used as informative markers for the discrimination of individuals with European, Sub-Saharan African and East Asian ancestry [15].

The solute carrier family 45 member 2 (*SLC45A2*), encodes a polypeptide that appears to be a transporter mediating melanin synthesis in skin pigmentation [20], [25]–[27]. The rs16891982 SNP displays differing population frequency distributions and these variants have been shown to be significantly associated with dark hair, skin, and eye pigmentation in Caucasian, Asians, African Americans, and Australian Aborigines populations [26]. Together with the SNP rs1426654 at *SLC24A5*, rs16891982 is known to explain a substantial proportion of skin pigmentation differences between Europeans and West Africans [22], [28].

Tyrosinase, encoded by the *TYR* gene in chromosome 11, is an essential enzyme for the synthesis of melanin [29], [30]. Defects of *TYR* are known to be the cause of oculocutaneous albinism type 1A (http://omim.org/entry/203100). The SNP rs1042602, is a C to A transversion within the coding region of the tyrosinase gene, leading to a S192Y mutation, which is associated with eye, hair and skin pigmentation in several populations [2], [11], [21].

### Prediction of color based on the genotypes of the four significant pigmentation-associated SNPs

We tried to assess whether the genotypes of the four SNPs that were significantly associated with color in the sample from Rio de Janeiro, could be used to make phenotypic predictions about which self-assessed color groups an individual belonged to. For that, we used the rs2555364-rs1426654 haplotypes, the genotypes at rs16891982 and rs1042602 genotypes and the graphical output of Structure software [31] to separate clusters among the individuals from our target population of Rio de Janeiro. The program assigned to each individual a value, which we called “Pigmentation Index (PI)” that show increasing values as we move sequentially from the White self-assessed color category to the Brown and Black ones (Figure 2). However, as the dot plot of PI values for the three color groups show (Figure 3) it is evident that the color groups had wide variance and that there was significant overlap between them, making it impossible to confidently predict self-assessed color group from SNP genotypes at an individual level.

Thus, neither biogeographical ancestry nor the pigmentation index (PI) calculated from the genotypes of rs1426654, rs16891982, rs2555364, rs1042602 were capable of predicting well the self-assessed choice of color group of individuals from Rio de Janeiro. Since such genotypes had been shown to be, at least in part, statistically independent of ancestry estimates, we tried to ascertain whether they together could generate a better prediction of self-assessed color, by using Principal Component Analysis (PCA) with three variables: Pigmentation index (PI), African ancestry and Amerindian ancestry (Figure 4). Again, by far, the largest variation was seen in the Brown category, especially along the first component.

In spite of the incomplete resolution of the three color categories, inspection of the PC1-PC2 biplot shows interesting features. From left to right in Figure 4 (moving progressively from a majority of Whites, to a majority of Browns, to a majority of Blacks) a spread is discernible, suggesting that variation along the second component becomes more relevant as we move rightwards. To try to understand the reason for this, we studied the non-parametric rank correlations (Spearman's rho) between the first and second component and the three variables used to calculate the principal components. Indeed, as shown in detail in Table S5, we find that PC1 is more highly correlated with Pigmentation Index (rho  = 0.885) and African ancestry (rho  = 0.835), while PC2 is more highly correlated with Amerindian ancestry (rho  = 0.714). Hence, is appears from Figure 4 that variation in Amerindian ancestry increases, as the amount of pigmentation and African ancestry increase. This in understandable historically, since Amerindian admixture has been more common with African slaves in the Brazilian past, than with Europeans, as shown in our study of mitochondrial DNA inheritance in Black individuals [32].

### The biological basis of self-assessment in color categories

The self-attribution of color is complex. It is influenced by the skin pigmentation, but also by other characteristics such as hair and eye pigmentation, facial features and family history, as well as extraneous factors that may range from sunlight exposure (Pena et al, 2011) to income level, social class and schooling [2], [5], [33].

Petrucelli (2007) notes that the Brown category has the additional complication of apparently designating a residual category in the racial classification system. Inside the Brown category, he distinguishes at least three categories: first, a group that has a phenotype that is perceived to be of African origin; secondly, a group that can be identified as predominantly of Amerindian descent and thirdly, a group that expresses an adhesion to a specific historical-geographical condition and does not actually constitutes a proper ethnic identification in the sense of physical appearance (Petrucelli, 2007). Thus, the Brown category poses an intrinsic classification problem. On the other hand, it is a quite important category, been chosen by 42% of Brazilians (http://www.sidra.ibge.gov.br/bda/tabela/listabl.asp?z=cd&o=4&i=P&c=3145).

Under the light of the above, it might be argued, *prima facie*, that the use of self-assessed color classification might not be the ideal parameter for a Brazilian study of the influence of genotype on pigmentation phenotype. Therefore, we should examine what would be the alternative approaches. One possibility would be the use of color classification by an external observer. However, this is equally subjective and different studies have shown that it does not lead to greatly different results when compared with self-classification [2], [33].

Another possibility might be the use of skin reflectance levels to measure the degree of pigmentation, as was used in the recent paper by Beleza et al [21]. On the basis of the genetic variance at four major pigmentation loci, the authors could explain 35% of the skin pigmentation variance, which is not much higher that what we have observed I this study using self-assessed color in Brazilians, a much more complex admixed population with three different ancestral roots. We should also consider that reflectance spectroscopy has a problem of lack of social relevance. When interacting socially, people do not make reflectance measures, but evaluate color according to the whole physical appearance of the individual. All considered, we find that the use of self-assessed color is still the best option for a Brazilian study.

Forensic scientists have been discussing the possibility of using genotypes at “color loci” to predict the pigmentation phenotypic features of perpetrators of felonies using DNA left in crime scenes [34], [35]. It would appear that independently of whether one uses skin reflectance or self-assessed color, this might not be a reliable forensic procedure in admixed populations, such as Cape Verde or Brazil.

In conclusion, in this study we could observe significant association of self-assessed color categories in Brazilians with genotypes at three genes *SLC24A5*, *SLC45A2* and *TYR*, which also have been found to be associated with skin color in other populations. Moreover, we used partial regression analysis to eliminate the confounding biogeographical ancestry as a covariate. We believe that this is novel and effective way to achieve genomic control for association studies in Brazilians. We also observed that neither genotypes at SNPs in the *SLC24A5*, *SLC45A2* and *TYR* loci, nor their combination with biogeographical ancestry in principal component analysis could predict self-assessed color in Brazilians at an individual level. However, significant correlations did emerge at group level, demonstrating that even though elements other than skin, eye and hair pigmentation influence self-assessed color in Brazilians, the sociological act of color self-classification is still substantially dependent of genotype at these four SNPs.

## Materials and Methods

### Ethics statement

The Research Ethics Committee of the Instituto Nacional do Câncer (INCA) approved in 2005 the protocol of this study, as part of a pharmacogenetic project, as well as the written informed consent form. In 2008 the same ethics Committee approved the enlargement of the study to its present format and carried forward the approval of the written informed consent form. The samples were anonymized after collection. Some of the DNA samples of the present study were analyzed in previous publication [1], [36], [37].

The use of the samples from the Laboratory of Genetics and Molecular Hematology of the Faculdade de Medicina da Universidade de São Paulo in 1997 and 2004, respectively, received the approvals CAPPesq 173/1997 and CAPPesq 543/2004 including the written Informed Consent form. The samples also were anonymized after collection. The individuals of the present study are a subset of a larger sample described in two previous studies [7], [38].

### Populations studied

We studied 455 unrelated Brazilians from two large cities (Rio de Janeiro and São Paulo) in the Southeast of Brazil as described in detail below. Color assignation was obtained by self-assessment in answer to the closed question “What is your color/race?” as done in the Brazilian census by the Instituto Brasileiro de Geografia e Estatística (IBGE). All subjects of this study described themselves as White, Brown or Black (in Portuguese, respectively, “Branco”, “Pardo” and “Preto”). These three color categories encompass 99.1% of the Brazilian population. No subjects in the study were self-classified as Indian (“Indígena”), Yellow (“Amarelo”) or did not declare a color (“Sem declaração”).

The INCA sample was made up of 243 unrelated, healthy individuals, all collected from blood donors, personnel and research students at the Instituto Nacional do Cancer (INCA). The enrolled individuals, were randomly chosen from within each color category, were self-identified as Whites (n = 82), Browns (n = 80) or Blacks (n = 81).

The São Paulo sample was made up of 212 unrelated healthy volunteer blood donors of the city of São Paulo collected as previously described by Bydlowski et al [7]. Besides self-classification, these individuals were also evaluated by phenotypic and genealogical criteria as follows: subjects were asked about their color group and those of their parents and grand-parents, according to their own definition, according to Census criteria. Phenotype analysis (facial characteristic and skin pigmentation in the axilla, a body region not exposed to the sun) was performed by the interviewer. In all cases there was concordance between the self-assessment and the phenotypic and genealogical criteria. Because of sample limitations, for this study we chose randomly only subjects who self-classified in one of two groups: Whites (n = 106) and Blacks (n = 106).

### Selection of SNPs

After analysis of human genetic variants associated with pigmentation of skin or eyes and tanning response, we selected for this study 15 SNPs, within nine different loci: rs1015362 (*ASIP*) [14], [39]; rs6058017 (*ASIP*) [18], [28]; rs12913832 (*HERC2*) [40], [41]; rs642742 (*KITLG*) [42]; rs1800401 (*OCA2*) [39], [43]; rs1800407 (*OCA2*) [43], [44]; rs12896399 (*SLC24A4*) [44], [45]; rs1426654 (*SLC24A5*) [18], [41]; rs2555364 (*SLC24A5*) [15], [46]; rs26722 (*SLC45A2*) [39], [47]; rs16891982 (*SLC45A2)* [18], [41]; rs1042602 (*TYR*) [14], [19]; rs1126809 (*TYR*) [14], [39]; rs1408799 (*TYRP1*) [14], [18] and rs2733832 (*TYRP1*) [42], [48].

### Genotyping

The chosen SNPs were genotyped using the real-time PCR *TaqMan* assay utilizing two differentially fluorescently labeled probes that permitted the detection of both alleles in a single reaction (Applied Biosystems INC, Foster City, CA, USA). The PCR primers and Taqman probes had been previously developed at Applied Biosystems and are listed in Supplementary Table S1 for each SNP studied. Assays were performed on an ABI 7900 HT *Fast PCR Real Time System* (*Applied Biosystems, Foster City, CA*), and the genotype assignments were conducted using the *TaqMan*™ *Genotyper Software*, in a 384-well format and using manufacturer's instructions.

The classification accuracy of each TaqMan assay was validated by cycle sequencing (forward and reverse) of PCR fragments containing the polymorphisms studied using the DYEnamicTM ET Dye Terminator Kit, (GE Healthcare) standard procedure and a MegaBACE™ 1000 sequencer (GE Healthcare). After the run in the MegaBACE sequencer, the electrofluorograms were visualized using *Sequencher*™ v 4.1.4 (*Gene Codes Corporation, Ann Arbor, USA*).

### Estimation of biogeographical ancestry

To estimate the relative proportion of Amerindian, European, and Sub-Saharan African ancestry for each sample from São Paulo we genotyped each sample using the following panel of 40-biallelic short insertion/deletion polymorphisms (indels): MID-1 (rs3917), MID-15 (rs4181), MID-17 (rs4183), MID-51 (rs16343), MID-89 (rs16381), MID-107 (rs16394), MID-131 (rs16415), MID-132 (rs16416), MID-150 (rs16430), MID-159 (rs16438), MID-170 (rs16448), MID-258 (rs16695), MID-278 (rs16715), MID-420 (rs140709), MID-444 (rs140733), MID-468 (rs140757), MID-470 (rs140759), MID-663 (rs1305047), MID-788 (rs1610874), MID-857 (rs1610942), MID-914 (rs1610997), MID-918 (rs1611001), MID-1002 (rs1611084), MID-1092 (rs2067180), MID-1100 (rs2067188), MID-1129 (rs2067217), MID-1291 (rs2067373), MID-1352 (rs2307548), MID-1428 (rs2307624), MID-1537 (rs2307733), MID-1549 (rs2307745), MID-1586 (rs2307782), MID-1642 (rs2307838), MID-1654 (rs2307850), MID-1759 (rs2307955), MID-1763 (rs2307959), MID-1847 (rs2308043), MID-1861 (rs2308057), MID-1943 (rs2308135) and MID-1952 (rs2308144). In this list, The MID number relates to the nomenclature of Weber et al. [49] and the rs numbers relate to dbSNP (http://www.ncbi.nlm.nih.gov/snp/).

This set of 40 indels had been previously validated as useful in ancestry estimation through the study of the HGDP-CEPH Diversity Panel, which is composed of 1,064 individuals from 52 different worldwide populations distributed in seven geographical regions [11]. The individual results have been deposited in the CEPH Genotype Database (http://www.cephb.fr/en/hgdp/main.php), from where they are available. The multiplex PCR assays and analysis of the indels were performed in an ABI 3130 Fluorescent Automatic Sequencer and analyzed using the *Sequencher*™ software (*Gene Codes Corporation, Ann Arbor, USA*).

To estimate the ancestry proportions from the indel genotyping results we used the *Structure* software [10]. This software uses multilocal genotypes to infer the structure of each population and to allocate probabilistically the proportion of genomic ancestry of individuals in different populations. As parameters we assumed the admixture model, correlated allele frequencies and used 100,000 burn-in steps followed by 900,000 Markov Chain Monte Carlo iterations. We used for reference populations, 158 Europeans, 125 Sub-Saharan Africans and 107 Amerindians of the HGDP-CEPH Diversity Panel, which had been typed as part of our previous studies with the same set of 40 indels [9].

The relative proportions of Amerindian, European and Sub-Saharan African biogeographical ancestries of the samples from Rio de Janeiro had previously been estimated [1].

### Statistical analysis

The regression analysis was performed to study the association of each polymorphism with self-classified skin color categories, the phenotype was numerically fixed as dependent variable, the genotypic data were fixed as independent variables and the ancestry values were fixed as covariates. Initially we made a numerical linear regression using Golden Helix SNP and Variation Suite software (SVS Version 7.2.2, (Golden Helix Inc., Bozeman, MT, USA). In addition, a partial regression model (from the same package) called Reduced x Full model was used with a type I error α of 5%, considering the Bonferroni correction. We introduced individual ancestry as a covariate in this last model in order to control spurious associations that may be the result of differences in the ancestral proportions (admixture). When the results (P values) of the two models were compared to determine which loci remain associated with the phenotype even after the correction for covariates (ancestry). For all SNPs numerical regression analyses were performed considering three genetic models: additive, dominant and recessive. The statistical significance was defined as p<0.05. P values were corrected by the Bonferroni adjustment for multiple comparisons tests.

## Supporting Information

File S1 **File includes Tables S1–S4.** Table S1: SNPs used in this study. Table S2: Estimative of allele frequencies and their standard errors for each SNP in each self-classified color category. Table S3: P values for association (after Bonferroni correction) using 15 SNPs related with pigmentation and self-assessed color in unrelated Brazilians from Rio de Janeiro under three models. Table S4: Locus *SLC24A5*: rs2555364-rs1426654 haplotype frequencies in the Rio de Janeiro population sample according to self-assessed color group.
(DOC)
