# Supplementary material for: Association of Genetic Variants with Self-Assessed Color Categories in Brazilians
Source: PLoS One. 2014 Jan 8;9(1):e83926. doi: 10.1371/journal.pone.0083926 (PMC3885524; doi:10.1371/journal.pone.0083926)
Supplement: File S1 — File includes Tables S1–S4. Table S1: SNPs used in this study. Table S2: Estimative of allele frequencies and their standard errors for each SNP in each self-classified color category. Table S3: P values for association (after Bonferroni correction) using 15 SNPs related with pigmentation and self-assessed color in unrelated Brazilians from Rio de Janeiro under three models. Table S4: Locus SLC24A5: rs2555364-rs1426654 haplotype frequencies in the Rio de Janeiro population sample according to self-assessed color group. (DOC) [file pone.0083926.s001.doc]

**Table S1** – SNPs used in this study

| Ref SNP | Chr^a^ | Position^b^ | SNP | Candidate Gene | Part-Number^c^ | Primer Name^d^ | Primer Sequence |
| --- | --- | --- | --- | --- | --- | --- | --- |
| rs26722 | 5 | 33963870 | C/T | *SLC45A2* | C__239057_1 | rs26722F | 5’-CAACAACAAAGAGCAAGAATAT-3’ |
|  |  |  |  |  |  | rs26722_2R | 5’-AGACTGTTGGGTACAGAATTCCA-3’ |
| rs16891982 | 5 | 33951693 | C/G | *SLC45A2* | C__2842665_10 | rs16891982_2F | 5’-TGATCAGGAACCCACTGATTC-3’ |
|  |  |  |  |  |  | rs16891982R | 5’-GATCCCTATAGTGCACACAAC-3’ |
| rs1408799 | 9 | 12672097 | C/T | *TYRP1* | C__1911864_10 | rs1408799_2F | 5’-TGAAAGGAATAAAACCACTCATCA-3’ |
|  |  |  |  |  |  | rs1408799R | 5’-GAGGACAGGACCATGTCTCT-3’ |
| rs2733832 | 9 | 12704725 | C/T | *TYRP1* | C__3119210_10 | rs2733832F | 5’-GGCTCATCTATTCCTGAATG-3’ |
|  |  |  |  |  |  | rs2733832_2R | 5’-TGATTTGGTTAGTCCCAGCA-3’ |
| rs1042602 | 11 | 88911696 | A/C | *TYR* | C__8362862_10 | rs1042602_2F | 5’-CTGGCAACTTCATGGGATTC-3’ |
|  |  |  |  |  |  | rs1042602R | 5’-ACCGCAACAAGAAGAGTCTA-3’ |
| rs1126809 | 11 | 89017961 | A/G | *TYR* | rs112689^1^ | rs1126809F | 5’-ATCTTTCCATGTCTCCAGATT-3’ |
|  |  |  |  |  |  | rs1126809_2R | 5’-TTCAGCAATTCCTCTGAAAGAA-3’ |
| rs642742 | 12 | 89299746 | A/G | *KITLG* | rs642742^1^ | rs642742_2F | 5’-TCAAAATCCTCGTGTGTTGAA-3’ |
|  |  |  |  |  |  | rs642742R | 5’-GGGTTTAAAGCATAGACTGTCT-3’ |
| rs12896399 | 14 | 92773663 | G/T | *SLC24A4* | C__3244615_10 | rs12896399_F | 5’-GGTTGCCAGATCTAGGAAATTG -3’ |
|  |  |  |  |  |  | rs12896399_R | 5’-CTGGAGCAAGACAATGGTACAG -3’ |
| rs1426654 | 15 | 48426484 | A/G | *SLC24A5* | C__2908190_10 | rs1426654_2F | 5-GCCTTCCCTCACCCTTTCTA-3’ |
|  |  |  |  |  |  | rs1426654R | 5’-CCAGCTAAGTTAGTGTTAACTTG-3’ |
| rs1800401 | 15 | 28260053 | C/T | *OCA2* | C__8866171_10 | rs1800401_2F | 5’-ACTGTTCATTGTCGGGTGGT-3’ |
|  |  |  |  |  |  | rs1800401R | 5’-AATATGATCAGCGCGTAGAC-3’ |
| rs1800407 | 15 | 28230318 | A/G | *OCA2* | C__8866200_10 | rs1800407F | 5’-CCCTGTTCTTAAAGTCACTAATG-3’ |
|  |  |  |  |  |  | rs1800407_2R | 5’-ATGCACCTGAGAATGGAACC-3’ |
| rs2555364 | 15 | 48419386 | C/G | *SLC24A5* | C__16038324_10 | rs2555364F | 5’-GACACAGCCGTCATATGCTC -3’ |
|  |  |  |  |  |  | rs2555364R | 5’-TTTGGTATTCCCCATCTCCA -3’ |
| rs12913832 | 15 | 28365618 | A/G | *HERC2* | C__30724404_10 | rs12913832F | 5’- TCTTAATTCAAAATGCCCCCAAGTA -3’ |
|  |  |  |  |  |  | rs12913832R | 5’- ACCACTGGTAGTTTTCTTTGCCAAT -3’ |
| rs1015362 | 20 | 32738612 | A/G | *ASIP* | C__7497804_10 | rs1015362_F | 5’-ACTAACCCCTCCTTTTCTTTGC -3’ |
|  |  |  |  |  |  | rs1015362_R | 5’-TTTTCTCGGAAATGGACGTTAT -3’ |
| rs6058017 | 20 | 32856998 | A/G | *ASIP* | C__2275334_10 | rs6058017F | 5’- AGGAGGCTTCGATGAAGAAAGT-3’ |
|  |  |  |  |  |  | rs6058017R | 5’-GAACTTTCGGGACCTTTACAGA -3’ |

^1^ In-house designed Taqman assays.

^a^ Chromossome.

^b^ Locus position.

^c^ Catalog Part Number of Taqman SNP genotyping assays.

^d^ Primers used to validate Taqman assays by DNA sequencing.

**Table S2**. Estimative of allele frequencyiesand THEIR standard errors for each SNP in each self-classified color category.

|  |  |  | Rio de Janeiro | | | | | | São Paulo (SP) | | | |
| --- | --- | --- | --- | --- | --- | --- | --- | --- | --- | --- | --- | --- |
| RefSNP | SNP | Allele | Whites | SE | Browns | SE | Blacks | SE | Whites | SE | Blacks | SE |
| rs26722 | C/T | C | 0.91 | 0.02 | 0.83 | 0.03 | 0.84 | 0.03 | - | - | - | - |
| rs16891982 | C/G | C | 0.35 | 0.04 | 0.58 | 0.04 | 0.82 | 0.03 | 0.24 | 0.03 | 0.89 | 0.02 |
| rs1408799 | C/T | C | 0.51 | 0.04 | 0.38 | 0.04 | 0.35 | 0.04 | - | - | - | - |
| rs2733832 | C/T | C | 0.59 | 0.04 | 0.70 | 0.04 | 0.76 | 0.03 | 0.56 | 0.04 | 0.84 | 0.03 |
| rs1042602 | A/C | A | 0.36 | 0.04 | 0.28 | 0.04 | 0.11 | 0.02 | 0.41 | 0.03 | 0.10 | 0.02 |
| rs1126809 | A/G | A | 0.31 | 0.04 | 0.30 | 0.04 | 0.29 | 0.04 | - | - | - | - |
| rs642742 | A/G | A | 0.43 | 0.04 | 0.33 | 0.04 | 0.58 | 0.04 | 0.23 | 0.03 | 0.68 | 0.03 |
| rs12896399 | G/T | G | 0.69 | 0.04 | 0.78 | 0.03 | 0.85 | 0.03 | 0.74 | 0.03 | 0.87 | 0.02 |
| rs1426654 | A/G | A | 0.83 | 0.03 | 0.56 | 0.04 | 0.28 | 0.04 | 0.93 | 0.02 | 0.25 | 0.03 |
| rs1800401 | A/G | A | 0.11 | 0.03 | 0.08 | 0.02 | 0.10 | 0.02 | - | - | - | - |
| rs1800407 | C/T | C | 0.94 | 0.02 | 0.95 | 0.02 | 0.97 | 0.01 | - | - | - | - |
| rs2555234 | C/G | C | 0.10 | 0.03 | 0.28 | 0.04 | 0.51 | 0.04 | 0.05 | 0.02 | 0.49 | 0.04 |
| rs12913832 | A/G | A | 0.43 | 0.04 | 0.28 | 0.04 | 0.27 | 0.04 | 0.66 | 0.03 | 0.09 | 0.02 |
| rs1015362 | C/T | C | 0.65 | 0.04 | 0.61 | 0.04 | 0.42 | 0.04 | 0.72 | 0.03 | 0.42 | 0.04 |
| rs6058017 | A/G | A | 0.77 | 0.04 | 0.68 | 0.04 | 0.51 | 0.04 | 0.84 | 0.03 | 0.52 | 0.04 |

SE - Standard Error of estimative of allele frequency

**Table S3.** P values for association (after Bonferroni correction) using 15 SNPs related with pigmentation and self-assessed color in unrelated Brazilians from Rio de Janeiro under three models

|  | | Additive | | | Dominant | | | Recessive | |
| --- | --- | --- | --- | --- | --- | --- | --- | --- | --- |
|  |  | | | Model | | |  | | |
|  | Additive | | | Dominant | | | Recessive | | |
| **RefSNP** | **NR** | | **CV** | **NR** | | **CV** | **NR** | | **CV** |
| rs26722 | 1.000E+00 | | 1.000E+00 | 9.824E-01 | | 6.995E-01 | 1.000E+00 | | 1.000E+00 |
| rs16891982 | 4.516E-17* | | 3.461E-09* | 7.288E-10* | | 8.150E-04* | 2.041E-13* | | 4.159E-09* |
| rs1408799 | 1.946E-01 | | 1.000E+00 | 1.000E+00 | | 1.000E+00 | 2.484E-01 | | 1.000E+00 |
| rs2733832 | 3.185E-02* | | 8.883E-01 | 7.510E-01 | | 1.000E+00 | 9.950E-03* | | 1.510E-01 |
| rs1042602 | 3.268E-06* | | 3.606E-02* | 3.169E-06* | | 2.141E-02* | 2.109E-01 | | 1.000E+00 |
| rs1126809 | 1.000E+00 | | 1.000E+00 | 1.000E+00 | | 1.000E+00 | 1.000E+00 | | 1.000E+00 |
| rs642742 | 1.610E-01 | | 1.000E+00 | 4.169E-02* | | 1.000E+00 | 1.000E+00 | | 1.000E+00 |
| rs12896399 | 5.235E-02 | | 1.000E+00 | 2.856E-01 | | 1.000E+00 | 1.319E-01 | | 1.000E+00 |
| rs1426654 | 4.038E-20* | | 2.086E-09* | 1.400E-19* | | 1.798E-10* | 2.525E-09* | | 5.209E-03* |
| rs1800401 | 1.000E+00 | | 1.000E+00 | 1.000E+00 | | 1.000E+00 | 1.000E+00 | | 1.000E+00 |
| rs1800407 | 1.000E+00 | | 1.000E+00 | 1.000E+00 | | 1.000E+00 | 1.000E+00 | | 1.000E+00 |
| rs2555234 | 6.123E-13* | | 6.667E-07* | 7.093E-11* | | 6.634E-06* | 7.499E-06* | | 7.721E-03* |
| rs12913832 | 1.039E-01 | | 1.000E+00 | 2.050E-04* | | 1.564E-01 | 1.000E+00 | | 1.000E+00 |
| rs1015362 | 8.444E-03* | | 1.000E+00 | 2.809E-01 | | 1.000E+00 | 8.474E-03* | | 1.000E+00 |
| rs6058017 | 2.878E-05* | | 5.349E-02 | 3.010E-04* | | 1.822E-01 | 1.682E-02* | | 5.688E-01 |

NR - Numeric full model regression.

CV - restricted model (partial correlation with ancestry as a covariate). Significant values (< 0.05) after Bonferroni correction are shown with an asterisk.

**Table S4**. Locus *SLC24A5:* rs2555364-rs1426654 haplotype frequencies in the Rio de Janeiro population sample according to self-assessed color group

|  | Whites | | Browns | | Blacks | |
| --- | --- | --- | --- | --- | --- | --- |
| Haplotype | E(freq) | S.E | E(freq) | S.E | E(freq) | S.E |
| CG | 0.00 | 0.00 | 0.28 | 0.00 | 0.49 | 0.01 |
| CA | 0.10 | 0.00 | 0.00 | 0.00 | 0.03 | 0.01 |
| GG | 0.16 | 0.00 | 0.17 | 0.00 | 0.23 | 0.01 |
| GA | 0.74 | 0.00 | 0.56 | 0.00 | 0.26 | 0.01 |

E(freq) - Haplotype frequency calculated by the EM algoritm

S.E - Standard Error of estimative of allele frequency
